# Immunological Insights in Equine Recurrent Uveitis

**DOI:** 10.3389/fimmu.2020.609855

**Published:** 2021-01-08

**Authors:** Roxane L. Degroote, Cornelia A. Deeg

**Affiliations:** Chair of Physiology, Department of Veterinary Sciences, LMU Munich, Munich, Germany

**Keywords:** retina, Mueller glia, vitreous, serum, lymphocyte, granulocyte, complement system, cytokines

## Abstract

Horses worldwide suffer from equine recurrent uveitis (ERU), an organ-specific, immune-mediated disease with painful, remitting-relapsing inflammatory attacks alternating with periods of quiescence, which ultimately leads to blindness. In course of disease, both eyes can eventually be affected and since blind horses pose a threat to themselves and their surroundings, these animals have to be killed. Therefore, this disease is highly relevant for veterinary medicine. Additionally, ERU shows strong clinical and pathological resemblance to autoimmune uveitis in man. The exact cause for the onset of ERU is unclear to date. T cells are believed to be the main effector cells in this disease, as they overcome the blood retinal barrier to invade the eye, an organ physiologically devoid of peripheral immune cells. These cells cause severe intraocular inflammation, especially in their primary target, the retina. With every inflammatory episode, retinal degeneration increases until eyesight is completely lost. In ERU, T cells show an activated phenotype, with enhanced deformability and migration ability, which is reflected in the composition of their proteome and downstream interaction pathways even in quiescent stage of disease. Besides the dysregulation of adaptive immune cells, emerging evidence suggests that cells of the innate immune system may also directly contribute to ERU pathogenesis. As investigations in both the target organ and the periphery have rapidly evolved in recent years, giving new insights on pathogenesis-associated processes on cellular and molecular level, this review summarizes latest developments in ERU research.

## Introduction

Insights in the pathogenesis of equine recurrent uveitis (ERU) have grown in recent years, especially on cellular and molecular level in periphery and in the eye itself. ERU is an organ-specific, immune-mediated disease, which is predominantly driven by CD4+ T cells (1–3). These are somehow activated in periphery and then manage to overcome the blood retinal barrier (BRB), entering the eye and causing intraocular inflammation (4). Since ERU has a remitting-relapsing character (2), increasing numbers of immune cells invade and accumulate in the eye with every inflammatory attack, gradually destroying their main target, the retina. These acute phases alternate with periods of quiescence and increase in severity as the disease progresses (5). Factors associating to ERU onset are still discussed and an exact cause is elusive to date. A genome-wide association study suggested a correlation of genetic variants influencing the expression of IL-17A and IL-17F with the development of ERU (6). This study also substantiated previous reports linking ERU to MHC class I haplotype ELA-A9 in warmblood horses (7). Direct evidence for a genetic component in ERU of warmblood horses, however, is still lacking. Disease prevalence among the horse population is high, ranging from 2%–25% in the US and 8%–10% in Europe (5, 8). ERU often affects both eyes and eventually leads to blindness if left untreated (9). These two factors, high prevalence and loss of sight, explain its importance for veterinary medicine, since blind horses have to be killed due to the threat they pose to their surroundings and themselves, which has a great personal but also economic impact on horse owners. Moreover, ERU has great potential to serve as a valuable spontaneous model for autoimmune uveitis in man, due to strong clinical and pathological similarities (3, 4, 10, 11).

In the past, several studies could show that the dysregulated immune response in ERU is directed against retinal autoantigens such as interphotoreceptor retinoid-binding protein (IRBP) and cellular retinaldehyde-binding protein (CRALBP) (4, 12), which, along with other factors, suggests presence of autoimmune reactions in ERU. These autoantigens not only remain stably expressed in advanced stages of ERU, when retinal architecture is drastically destructed (13), IRBP can also induce experimental uveitis in the horse itself, which shows close similarity to the spontaneous disease on clinical, cellular and molecular level (4, 14). Moreover, studies on ERU horses firstly provided CRALBP as novel autoantigen (12), which was subsequently proven to also have high prevalence in human patients with autoimmune uveitis (15). As shown in previous studies, the recognition of these autoantigens and the subsequent spreading of epitope recognition to a new determinant may be the cause for the relapsing character of ERU (12, 16). This process known as epitope spreading describes the reaction of immune cells against the initial epitope of an ERU autoantigen, which then redirects against a different, but structurally similar epitope, re-initializing an inflammatory response after the initial inflammation has subsided (17, 18).

In addition to the retinal-autoantigen specific T cells driving ERU from periphery, further studies suggested that the retina itself promotes the intraocular inflammatory processes through active abundance downscaling of proteins which protect the maintenance of the blood-retina-barrier (19) and through secretion of the pro inflammatory cytokine interferon gamma (IFNγ) by retinal Mueller glial cells (20). Since then, research has advanced on the target organ of ERU as well as the cellular and molecular components in periphery. This review summarizes the latest immunological insights in ERU pathogenesis.

## Periphery

### Adaptive Immune System

Although the concept of immune privilege is increasingly discussed since its initial description (21, 22), the eye is considered to be an immune privileged organ (23, 24), and is therefore usually devoid of immune cells form peripheral blood stream. In ERU, however, we find major cell infiltrates in the eye (2, 3, 25–27), which mainly consist of CD4+ lymphocytes (2, 27). To date, the exact mechanisms enabling these autoreactive cells to overcome the BRB in ERU are still elusive. Experimental autoimmune uveitis (EAU) is a valuable tool to investigate these mechanisms, not only in common rodent models (28–31), but to a certain extent also in the horse (4). Immunization of healthy horses with the retinal autoantigen IRBP mimics the spontaneous disease through the formation of auto-aggressive, IRBP specific T cells, which leave the peripheral blood stream to invade the eye directly prior to a uveitic attack (4). These cells show distinct protein changes before immunization, during the inflammatory phase and in quiescent episodes (14), which points to a highly dynamic functional phenotype depending on the stage of disease. During quiescence of this IRBP induced equine EAU, protein abundance changes in immune cells show many similarities to those observed in freshly obtained cells from horses in quiescent stage of ERU (14), underlining the close resemblance of the spontaneous and the induced disease in the horse. Although this type of induced horse model may allow more in depth investigations on ERU pathomechanisms, which is especially interesting for assessment of protein and cellular dynamics throughout the different stages of ERU, it is less suitable for large-scale experiments comparable to those performed on the several existing rodent models for autoimmune uveitis.

Analysis of primary T cells from horses with spontaneously occurring ERU revealed distinct proteome changes, mainly of proteins with a role in cell adhesion, cell migration and regulation of cell shape (32–34). In transmigration experiments, CD4+ T cells from ERU horses showed increased migration rates, which was linked to increased expression of the protein formin-like 1 (FMNL1) in the membranes of these cells (33). As shown in a new FMNL1 knock-out mouse model, FMNL1 deficiency impedes extravasation and trafficking of T cells to sites of inflammation (35), further undermining the importance of this protein and its functional effects in ERU pathogenesis. Furthermore, lymphocytes from ERU horses displayed significantly higher cell motility, cell speed and directness toward selected chemoattractants, especially IFNγ, and toward the ERU autoantigen CRALBP, in live-cell imaging experiments (36), which was associated to lower abundance of the protein septin 7 in these cells (32, 36). Previous studies on septin 7-depleted murine CD4+ T cells (clone D10.G4, as well as primary T cells from DO11.10 TCR transgenic mice), showed less rigidity and aberrant cell morphology in these depleted cells, resulting in the ability to migrate through narrow pores (37), which was also hypothesized for ERU (32), but never directly proven. Identification of interactors of septin 7, most importantly dedicator of cytokinesis 8 (DOCK8), which was decreased in ERU (34), pointed to impaired immunity and increased risk for recurrent inflammation, as suggested in studies on human cells from patients with DOCK8 deficiency (38). Furthermore, increased expression of DOCK8 interacting protein integrin-linked kinase (ILK) was functionally associated to a decreased apoptosis rate in ERU cells (34). Combined results from these *in vitro* experiments - increased life span, increased migratory reactivity toward chemokines and autoantigens and enhanced migration ability of ERU lymphocytes - underlines the dysregulated nature of these cells in ERU. Although the transfer of these interpretations from *in vitro* experiments to *in vivo* mechanisms needs to be assessed with care, these insights further support the role of CD4+ T cells as key players in ERU pathogenesis.

Recent studies showed that, although percentage of CD3+, CD8+, and CD4+ lymphocytes does not differ between healthy horses and those with ERU, only the disease-driving CD4+ T cells have an activated phenotype in ERU horses (39). These cells show significantly increased expression levels of IFNγ and decreased expression of IL‐10, indicating Th1 immune response (39). This supports previous findings of increased levels of IFNγ as well as cytokine Interferon gamma-induced protein 10 (IP-10) in serum of ERU horses (40). A further known immune response in autoimmune uveitis in man as well as EAU in rodents is mediated through Th17 cells (41). In ERU, the exact role of Th17 cells has not been established to date, however, detection of cytokines IL‐6, IL‐17, and IL‐23 *via* crossreactive anti-human antibodies in histological sections of the iris and the ciliary body of ERU horses provide first indications pointing toward a contribution of these cells to ERU pathogenesis (42). Similar conclusions could be drawn from *M. tuberculosis* (strain H37Ra) induced experimental uveitis in horses, where high levels of IL-17 were detected in aqueous humor and vitreous *via* Enzyme-linked Immunosorbent Assay (ELISA) (43).

Contrary to the Th1 and/or Th17 immune response inducing and maintaining inflammation in the uveitic eye, remission of each inflammatory bout in autoimmune uveitis and EAU can be associated to T regulatory cells (Treg) (44–46). During an acute inflammatory uveitic attack, these cells show decreased abundance in peripheral blood as opposed to phases of quiescence, suggesting a crucial role in the remitting-relapsing character of autoimmune uveitis (47, 48). These differences, however, could not be detected in peripheral blood of ERU horses compared to healthy controls (39). To date, no direct evidence for Tregs in the equine eye could be found. Nevertheless, the role of Treg in periodic remission of ERU is highly probable and thus merits further investigations.

Although an autoreactive Th1 response against retinal antigens is widely supported as the key process in ERU pathogenesis, one pivotal question remains: how can peripheral T cells be primed against tissue specific self-antigens which are “hidden” in an immune privileged organ? Emerging investigations on autoimmune uveitis suggest that commensal microbiota, if dysregulated (dysbiosis), may show sequence similarities with self-antigens (49, 50). This in turn may induce T cell cross-activation and priming against retina-specific antigens, triggering autoimmune reactions (49, 50). Other studies suggest, that a healthy gut microbiome is needed for prevention of autoimmunity through Treg homeostasis and that dysbiosis leads to changed or insufficiently effective Treg populations (51, 52). In ERU, the involvement of commensal microbiota has not been proven to date, however it is highly likely to play a possible role.

Equine T cell activation can be markedly reduced by co-incubation with adipose‐derived equine mesenchymal stem cells (MSC) *in vitro*, resulting in downscaling of CD25 as well as intracellular IFNγ, IL-10, and FoxP3 (39). This feature of MSC might be useful for the development of new therapeutic approaches in ERU (53), as suggested by in equine immune-mediated keratitis, where subconjunctival injection of autologous MSCs improved clinical signs of disease (54). Although clinical application of this therapeutic strategy needs more in-depth validation, it might have the potential of broadening the currently applied methods of medical and surgical therapy, which classically comprise the systemical and topical application of immunosuppressive and anti-inflammatory medication, placement of a suprachoroidal cyclosporine sustainded-release device or pars plana vitrectomy (9, 55, 56).

### Innate Immune System

The role of innate immune cells in autoimmune uveitis has been intensely investigated in the eyes of rodent EAU models (57–60) and infiltration of these cells in eyes of ERU horses, although to a lesser extent, has also been observed (3). The involvement of monocytes, which might promote pro-inflammatory behavior, as well as destruction of the targeted retinal cells in ERU could be shown by expression of CD68 on infiltrating peripheral immune cells in the diseased retina (61). Monocytes are major mediators of tissue damage in EAU (62, 63) and are potent antigen-presenting cells (64), which makes their role in ERU especially interesting, particularly in regard to their interaction with specific retinal cells (65). Further indication of participation of innate immune cells in ERU was proven by identification of decreased talin 1 abundance, a key integrin regulator in leukocytes, and its interacting proteins in low-density-neutrophils which were obtained as byproduct of lymphocyte isolation (25, 66). Targeted investigations of pure granulocyte fractions of healthy and ERU horses revealed significant proteome changes in diseased state, which associated to MHC-I-mediated antigen presentation, RAF/MAP kinase cascade, and neutrophil degranulation (67). Interestingly, increased neutrophil degranulation was also described in other T-cell driven autoimmune diseases, such as multiple sclerosis, where it is linked to a generally pre-activated state of these cells (68). Moreover, neutrophil degranulation relates to deviant equine granulocyte proteome after *in vitro* stimulation (69), underlining a latent state of activation of granulocytes in ERU, even in quiescent stage of disease (67). The fact that neutrophils from ERU horses more readily perform NETosis supports these findings (70). The exact role of innate immune cells and their timing in and impact on ERU pathogenesis, however, is still not completely understood to date and needs more in-depth investigation.

Implication of a role of the complement system was shown by the identification of several complement factors and split factors and their increased expression in ERU sera and eyes (19, 61, 71). Namely complement factor B, as well as C3 derived split products C3d and iC3b showed highly increased abundance, indicating strong activation of the complement system not only in the peripheral blood stream but also locally in the eye itself (61, 71). Although the exact source of these complement components in ERU eyes is still unclear, they might be macrophage-derived, as suggested by studies on human retinas from patients with age-related macular degeneration (AMD) and *in vivo* retina analyses of rodent models for AMD (72). Other studies on C57BL/6 mice showed that complement factor B is constitutively expressed by RPE cells and this expression is positively regulated by inflammatory cytokines in the human RPE cell line ARPE19 (73), a process which might also apply to the increased occurrence in ERU eyes. Deviant regulation of the complement system is involved in the pathogenesis of various ocular diseases, including autoimmune uveitis (74, 75) and the severity of experimental autoimmune uveitis drastically decreases in complement-receptor deficient rodent models (76–78). Moreover, EAU can be effectively suppressed in C57BL/6 mice when activation of complement *via* the alternative pathway is blocked through complement receptor CRIg-Fc. Since complement factor B is exclusive to the alternative pathway, increased intraocular levels in EAU suggest a role of this pathway in disease pathogenesis (79). Despite the high abundance of complement factor B in uveitic horse eyes, indicating a possible contribution of the alternative pathway to ERU pathogenesis, this correlation has not yet been assessed.

### Serum Proteins

In the last decade, proteomic studies on sera of ERU diseased horses revealed substantial differences compared to sera of healthy horses (80). Due to breakdown of the BRB in course of multiple uveitic attacks, several of these proteins can also be detected inside the eye (3, 71, 81, 82).

Among these differentially expressed proteins, kininogen was identified with decreased abundance in sera of ERU horses (71). Contrary to this, kininogen showed increased levels in vitreous and retina in ERU, whereas it was not detectable in healthy eyes (71). Kininogen promotes angiogenesis and neovascularization (83), a process that plays a significant role in the pathogenesis of autoimmune uveitis (71). It is also a part of the kallikrein-kinin system, which promotes integrity loss of barrier systems, such as the blood-brain-barrier in a mouse model of multiple sclerosis (84). Increased vitreal and retinal kininogen levels combined with a decrease in serum of ERU horses therefore points to disruption of the BRB in course of disease. IgM levels were also increased in ERU sera compared to healthy horses, suggesting a recent immune response pattern prior to blood sampling (71).

Decreased levels of pigment epithelium derived factor (PEDF) could be shown in sera of ERU horses (82), which was also observed in the retina and the vitreous of these animals in earlier studies (19, 20). PEDF was also decreased in plasma and retina from rats with endotoxin-induced uveitis, describing PEDF as a negative acute-phase protein (85). Furthermore, PEDF plays an important role in the viability of rat and mouse retinal cells (86, 87), the protection of human retinal pigment epithelium cells (ARPE-19 cell line) against oxidative stress (88) and the protection of tight junction proteins in rat eyes (85). Therefore, the decrease of PEDF in periphery as well as the target organ suggests increased permeability of the blood retinal-barrier.

## Target Organ

### Retinal Pigment Epithelium

Increased BRB permeability in ERU is supported by studies on the outer BRB, which physiologically maintains ocular immune privilege through disconnection of the inner eye from peripheral blood-derived immune cells (24). The outer BRB is formed by retinal pigment epithelium (RPE) cells, which play a crucial role in the mainly avascular retina of the horse, since they maintain homeostasis of retinal cells (89). This is also reflected in the RPE surface proteome, which strongly associates to transport processes (90). Furthermore, horses with ERU display a distinctly changed RPE membrane protein repertoire in ERU (91). A significant decrease of peripherin 2, a protein that is associated with membrane fusion processes, in RPE cells of ERU horses is thought to provoke disruption of the cell-to-cell junctions which are physiolocically maintained through these cells (92), which in turn points to increased permeability of the outer BRB. As also hypothesized for PEDF (19, 20, 82), this might promote integrity loss of the BRB. Although loss of barrier function of the BRB and leukocyte infiltration in course of ERU is evident (4, 27, 91, 92), the exact chronological order of these events is still not completely clear. Integrity loss of the BRB may occur either as an initial process which subsequently facilitates migration of peripheral immune cells into the eye, or as a secondary effect in response to leukocyte recruitment. The latter could be shown in an IRBP-induced B10.RIII mouse EAU model, where Evans blue dye, which was injected shortly before killing of the mice, could be observed leaking into the retinal parenchyma only after intravascular sticking and extravasation of blood-derived leukocytes from retinal venules took place (93). Further studies on murine EAU models implied that BRB breakdown might be a result of several consecutive steps, actively triggered by intravascular adherence of primed lymphocytes, followed by changed expression of adhesion molecules and chemokine receptors in the vascular epithelium and reduced shear stress in retinal veins prior to infiltration (93–97). Interestingly, adoptive transfer of EAU in lewis rats could show that antigen specificity for retinal antigens is not a prerequisite for migration of activated T cells through the BRB, however, for the induction of actual intraocular inflammation, it is indispensable (98).

### Retina and Vitreous

After infiltrating the eye, peripheral immune cells accumulate in the iris, ciliary body and retina of horses with ERU (4, 27). Although the vast majority of the infiltrating cells were identified as CD4+ T cells (2, 27), pure granulocyte infiltrates can be observed in few spontaneous ERU and IRBP induced cases (3, 4). This is in stark contrast to the composition of the cellular infiltrate in IRBP-induced murine EAU models, where a more heterogeneous cell infiltrate can be observed, containing around 30% CD4+ T cells and a high percentage of macrophages (99, 100). This points to different molecular pathways and cellular processes in EAU and ERU, underlining the great variability of disease pathogenesis between species, which needs to be kept in mind while interpreting associated data. In the base of the ciliary body and the iris of ERU horses, the infiltrated cells organize into lymphoid follicle nodules, which predominantly comprise CD3+ T cells (26, 27). In the retina, we mostly see scattered areas of T cell infiltration, especially near the ora ciliaris retinae and the optic disc, but occasional lymphoid follicle formation is also observed (27). Interestingly, the majority of infiltrated CD4+ lymphocytes in the ERU retina also show surface expression of CD166, a molecule associated with activation and transmigration of T cells into inflamed tissues (26). Presence of phosphorylated signal transducer and activator of transcription (STAT) proteins 1 and 5 in these cells underline the role of activated Th1 cells in ERU but also indirectly point toward inhibition of the Th17 pathway (26). Detection of IL‐6, IL‐17, and IL‐23 in cell infiltrates of the ciliary body and iris, on the other hand, indirectly suggests that a Th17 mediated immune reaction might also present in ERU eyes (42). In rodent models, EAU can develop either as a Th1 or a Th17 response depending on the model and the antigen used for induction, showing the importance of both T cell subpopulations in disease pathogenesis (41, 101–104). In ERU, on the other hand, the important role of Th1 lymphocytes as effector cells is well known (2, 3, 36), the exact impact of Th17 cells on disease pathogenesis, however, merits further investigations.

Through membrane proteome analyses of equine retinae, numerous differentially expressed proteins were identified in ERU, several of which were associated to retinal Mueller glia cell derived proteins (105). Retinal Mueller glia cells, the main macroglial cells of the retina, are indispensable for the maintenance of retinal function and integrity (106). Apart from their proinflammatory role in ERU (20) and in murine EAU (65), these cells display a gliotic phenotype, which presents with morphological changes and impaired functionality in diseased retinae (12, 20, 105, 107). One of the important functions of these cells is the regulation of potassium and water homeostasis in the retina (108, 109), which shows severe misbalance in ERU (105, 107). This is mirrored in changed abundance and distribution pattern of potassium channels Kir4.1 and Kir2.1 as well as water channels AQP4, AQP5, and AQP11 which is hypothesized to contribute to impaired Mueller cell function and intracellular fluid regulation and, subsequently, retinal edema (105, 107, 110), a severe complication of ERU (27). Furthermore, in ERU, dysfunctional RMG are connected to decreased secretion of Wnt signaling inhibitors DKK3 and SFRP2, which point to a regulatory role of Wnt signaling in ERU (111), similar to other autoimmune diseases (112, 113). These insights on RMG support the role of these cells as prime responders to autoimmune triggers in ERU, promoting inflammatory processes and increasing severity of disease pathogenesis.

Additionally, severe extracellular matrix (ECM) remodeling in ERU was suggested through the identification of the proteins fibronectin and osteopontin (OPN) (105, 114). Fibronectin is an important constituent of the vertebrate ECM, mediates cell-ECM interactions (115) and also plays a role in adhesion and migration of cultured rat Mueller cells (116). Since fibronectin expression in Mueller cell endfeet might contribute to the attachment of the retina to the vitreous body, the changed distribution of fibronectin in ERU retinae points to impaired Mueller cell adhesion (114). OPN is a multifunctional protein with pro-inflammatory as well as neuroprotective properties (117, 118). In EAU mice, an increased abundance of OPN correlates with severity of inflammation (119), whereas Mueller glia-derived OPN promotes photoreceptor survival in the Pde6brd1 mouse model of retinal degeneration (120). OPN also shows neuroprotective properties in porcine Mueller glia cells *in vitro* (121). Decreased expression of OPN in vitreous and retina might therefore point to reduced neuroprotection in ERU retinae (114). ERU-associated lack of neuroprotection in the retina was underlined by the identification of decreased levels and decreased activity of tissue inhibitor of metalloproteinases (TIMP)-2 in retina and vitreous (122). TIMP2 influences the activity of metalloproteinases (MMP) (123), which modulate cell-cell and cell-ECM interactions (124). It also has neuroprotective properties (125) and can inhibit migration of cells over physiological barriers (126). Hence, decrease in TIMP2 activity might promote the inflammatory responses in ERU. It has been shown that migrating immune cells, especially Th1 cells, increase their MMP2 and MMP9 expression to overcome blood-tissue barriers (127) and that inhibition of these MMPs ameliorates experimental autoimmune uveitis in rodent models (128, 129). Interestingly, infiltrating cells in ERU retinae stained highly positive for MMP9 (122).

Apart from high levels of IgG in eyes of ERU horses (3), ERU vitreous also contains various immunoglobulins of the subclass M (IgM), which show a broad reaction pattern against retinal proteins (81), whereas healthy eyes contain no IgM (or IgG). Since IgM is an activator of the complement system and complement has been proven to be highly present in the inner eye of ERU horses (61, 111), the presence of IgM in the ERU vitreous further implies involvement of innate immune system components in this T cell driven autoimmune disease. Further screening of retinal protein with vitreous from ERU horses provided potential retinal autoantigens which are targeted by IgM autoantibodies (81). One of these IgM targets was identified as neurofilament medium (NF-M) (81, 130). Interestingly, IgG response to NF-M was merely detectable, supporting the presence of a thymus-independent immune reaction with prolonged persistence of IgM response toward NF-M and lack of IgM/IgG switch (130). In the retina, on the other hand, NF-M showed decreased expression (130). The combination of active downscaling in damaged RMG and release of NF-M fragments into the adjacent vitreous might cause this combination of high vitreal occurrence and low retinal levels of NF-M (130). A further novel membrane-bound autoantigen, synaptotagmin-1, was identified with decreased expression in ERU (131), which might be a result of active downscaling of this protein in the retina through impaired neurotransmitter release in ERU. Since Synaptotagmin-1 is also expressed in the pineal gland, and pinealitis is known to concurrently develop in ERU horses (132), synaptotagmin-1 might play a role in pineal immunopathology in ERU (131). Whether these IgM-targeted retinal antigens qualify as autoantigen targets for ERU, however, remains to be proven in further studies.

## Conclusions

Overall, these novel immunological insights in ERU pathogenesis point to a complex interplay of several dysregulated mechanisms, which, on the one hand, can be linked to changes in cellular and humoral components of the immune system, such as a deviant functional phenotype of T cells with increased migratory ability and a decreased apoptosis rate, latently activated granulocytes and involvement of the alternative pathway of the complement system. On the other hand, this dysregulation points to a pivotal pro-inflammatory role of retinal cells with critically impaired function in the target organ itself. Although these findings shed more light on disease mechanisms, the interaction of these dysfunctional molecular mechanisms driving ERU as well as their exact individual role and timing in ERU pathogenesis needs further assessment. Establishment of a Mueller glia cell line (133) and characterization of cultured RPE cells (90) provide valuable tools for more in depth functional examinations of ERU pathology. Additionally, the possibility of commensal microbiota involvement in ERU onset needs to be addressed.

## Author Contributions

CD conceived the outline of the manuscript. CD and RD wrote the manuscript. All authors contributed to the article and approved the submitted version.

## Funding

Work from research group Deeg mentioned in this review was supported by grants from the Münchener Universitätsgesellschaft 31697 (to RD) and the Deutsche Forschungsgemeinschaft DFG DE 719/4-3 (to CD).

## Conflict of Interest

The authors declare that the research was conducted in the absence of any commercial or financial relationships that could be construed as a potential conflict of interest.

## References

[1] Romeike A Brugmann M Drommer W Immunohistochemical studies in equine recurrent uveitis (ERU). Vet Pathol (1998) 35(6):515–26. 10.1177/030098589803500606 9823593

[2] Gilger BC Malok E Cutter KV Stewart T Horohov DW Allen JB Characterization of T-lymphocytes in the anterior uvea of eyes with chronic equine recurrent uveitis. Vet Immunol Immunopathol (1999) 71(1):17–28. 10.1016/S0165-2427(99)00082-3 10522783

[3] Deeg CA Kaspers B Gerhards H Thurau SR Wollanke B Wildner G Immune responses to retinal autoantigens and peptides in equine recurrent uveitis. Invest Ophthalmol Vis Sci (2001) 42(2):393–8. 11157872

[4] Deeg CA Thurau SR Gerhards H Ehrenhofer M Wildner G Kaspers B Uveitis in horses induced by interphotoreceptor retinoid-binding protein is similar to the spontaneous disease. Eur J Immunol (2002) 32(9):2598–606. 10.1002/1521-4141(200209)32:9<2598::AID-IMMU2598>3.0.CO;2-# 12207344

[5] Gerding JC Gilger BC Prognosis and impact of equine recurrent uveitis. Equine Vet J (2016) 48(3):290–8. 10.1111/evj.12451 25891653

[6] Kulbrock M Lehner S Metzger J Ohnesorge B Distl O A genome-wide association study identifies risk loci to equine recurrent uveitis in German warmblood horses. PloS One (2013) 8(8):e71619. 10.1371/journal.pone.0071619 23977091 PMC3743750

[7] Deeg CA Marti E Gaillard C Kaspers B Equine recurrent uveitis is strongly associated with the MHC class I haplotype ELA-A9. Equine Vet J (2004) 36(1):73–5. 10.2746/0425164044864651 14756375

[8] Spiess BM Equine recurrent uveitis: the European viewpoint. Equine Vet J Suppl (2010) 37:50–6. 10.1111/j.2042-3306.2010.tb05635.x 20939167

[9] McMullen RJ Jr. Fischer BM Medical and Surgical Management of Equine Recurrent Uveitis. Vet Clin North Am Equine Pract (2017) 33(3):465–81. 10.1016/j.cveq.2017.07.003 28985983

[10] Deeg CA Hauck SM Amann B Pompetzki D Altmann F Raith A Equine recurrent uveitis–a spontaneous horse model of uveitis. Ophthalmic Res (2008) 40(3-4):151–3. 10.1159/000119867 18421230

[11] Neutzner RV Jager M Friedburg C Deeg CA Lorenz B [Blind spot enlargement syndrome in acute zonal occult outer retinopathy with detection of autoantibodies against the retinal antigens CRALBP and S-Ag]. Ophthalmologe (2011) 108(11):1045–9. 10.1007/s00347-011-2406-x 21904838

[12] Deeg CA Pompetzki D Raith AJ Hauck SM Amann B Suppmann S Identification and functional validation of novel autoantigens in equine uveitis. Mol Cell Proteomics (2006) 5(8):1462–70. 10.1074/mcp.M500352-MCP200 M500352-MCP200 [pii]. 16690753

[13] Deeg CA Hauck SM Amann B Kremmer E Stangassinger M Ueffing M Major retinal autoantigens remain stably expressed during all stages of spontaneous uveitis. Mol Immunol (2007) 44(13):3291–6. 10.1016/j.molimm.2007.02.027 S0161-5890(07)00096-X [pii]. 17467057

[14] Hauck SM Lepper MF Hertl M Sekundo W Deeg CA Proteome Dynamics in Biobanked Horse Peripheral Blood Derived Lymphocytes (PBL) with Induced Autoimmune Uveitis. Proteomics (2017) 17(19):1–5. 10.1002/pmic.201700013 28846213

[15] Deeg CA Raith AJ Amann B Crabb JW Thurau SR Hauck SM CRALBP is a highly prevalent autoantigen for human autoimmune uveitis. Clin Dev Immunol (2007) 2007:39245. 10.1155/2007/39245 18317528 PMC2246040

[16] Deeg CA Amann B Raith AJ Kaspers B Inter- and intramolecular epitope spreading in equine recurrent uveitis. Invest Ophthalmol Vis Sci (2006) 47(2):652–6. 10.1167/iovs.05-0789 47/2/652 [pii]. 16431964

[17] Vanderlugt CJ Miller SD Epitope spreading. Curr Opin Immunol (1996) 8(6):831–6. 10.1016/s0952-7915(96)80012-4 PMC7135770 8994863

[18] Ujiie H Yoshimoto N Natsuga K Muramatsu K Iwata H Nishie W Immune Reaction to Type XVII Collagen Induces Intramolecular and Intermolecular Epitope Spreading in Experimental Bullous Pemphigoid Models. Front Immunol (2019) 10:1410. 10.3389/fimmu.2019.01410 31275329 PMC6593113

[19] Deeg CA Altmann F Hauck SM Schoeffmann S Amann B Stangassinger M Down-regulation of pigment epithelium-derived factor in uveitic lesion associates with focal vascular endothelial growth factor expression and breakdown of the blood-retinal barrier. Proteomics (2007) 7(9):1540–8. 10.1002/pmic.200600795 17407186

[20] Hauck SM Schoeffmann S Amann B Stangassinger M Gerhards H Ueffing M Retinal Mueller glial cells trigger the hallmark inflammatory process in autoimmune uveitis. J Proteome Res (2007) 6(6):2121–31. 10.1021/pr060668y 17444670

[21] Forrester JV Xu H Good news-bad news: the Yin and Yang of immune privilege in the eye. Front Immunol (2012) 3:338. 10.3389/fimmu.2012.00338 23230433 PMC3515883

[22] Forrester JV McMenamin PG Dando SJ CNS infection and immune privilege. Nat Rev Neurosci (2018) 19(11):655–71. 10.1038/s41583-018-0070-8 30310148

[23] Streilein JW Takeuchi M Taylor AW Immune privilege, T-cell tolerance, and tissue-restricted autoimmunity. Hum Immunol (1997) 52(2):138–43. 10.1016/S0198-8859(96)00288-1 S0198-8859(96)00288-1 [pii]. 9077562

[24] Taylor AW Ng TF Negative regulators that mediate ocular immune privilege. J Leukoc Biol (2018) 103(6)1179–87. 10.1002/JLB.3MIR0817-337R PMC6240388 29431864

[25] Degroote RL Hauck SM Treutlein G Amann B Frohlich KJ Kremmer E Expression Changes and Novel Interaction Partners of Talin 1 in Effector Cells of Autoimmune Uveitis. J Proteome Res (2013) 12(12):5812–9. 10.1021/pr400837f 24144192

[26] Kleinwort KJ Amann B Hauck SM Feederle R Sekundo W Deeg CA Immunological Characterization of Intraocular Lymphoid Follicles in a Spontaneous Recurrent Uveitis Model. Invest Ophthalmol Vis Sci (2016) 57(10):4504–11. 10.1167/iovs.16-19787 27571017

[27] Deeg CA Ehrenhofer M Thurau SR Reese S Wildner G Kaspers B Immunopathology of recurrent uveitis in spontaneously diseased horses. Exp Eye Res (2002) 75(2):127–33. 10.1006/exer.2002.2011 12137758

[28] Caspi RR Roberge FG McAllister CG el-Saied M Kuwabara T Gery I T cell lines mediating experimental autoimmune uveoretinitis (EAU) in the rat. J Immunol (1986) 136(3):928–33. 2416842

[29] Palestine AG Mc Allister C Carter C Keenan AM Vistica B Gery I Lymphocyte migration in the adoptive transfer of EAU. Invest Ophthalmol Vis Sci (1986) 27(4):611–5. 3485614

[30] Caspi RR Th1 and Th2 responses in pathogenesis and regulation of experimental autoimmune uveoretinitis. Int Rev Immunol (2002) 21(2-3):197–208. 10.1080/08830180212063 12424843

[31] Caspi RR Chan CC Fujino Y Najafian F Grover S Hansen CT Recruitment of antigen-nonspecific cells plays a pivotal role in the pathogenesis of a T cell-mediated organ-specific autoimmune disease, experimental autoimmune uveoretinitis. J Neuroimmunol (1993) 47(2):177–88. 10.1016/0165-5728(93)90028-w 8370769

[32] Degroote RL Hauck SM Amann B Hirmer S Ueffing M Deeg CA Unraveling the equine lymphocyte proteome: differential septin 7 expression associates with immune cells in equine recurrent uveitis. PloS One (2014) 9(3):e91684. 10.1371/journal.pone.0091684 24614191 PMC3951111

[33] Degroote RL Uhl PB Amann B Krackhardt AM Ueffing M Hauck SM Formin like 1 expression is increased on CD4+ T lymphocytes in spontaneous autoimmune uveitis. J Proteomics (2017) 154:102–8. 10.1016/j.jprot.2016.12.015 28057602

[34] Schauer M Kleinwort KJH Degroote RL Wiedemann C Kremmer E Hauck SM Interaction of septin 7 and DOCK8 in equine lymphocytes reveals novel insights into signaling pathways associated with autoimmunity. Sci Rep (2018) 8(1):12332. 10.1038/s41598-018-30753-7 30120291 PMC6098150

[35] Thompson SB Sandor AM Lui V Chung JW Waldman MM Long RA Formin-like 1 mediates effector T cell trafficking to inflammatory sites to enable T cell-mediated autoimmunity. Elife (2020) 9:1–27. 10.7554/eLife.58046 PMC7308091 32510333

[36] Wiedemann C Amann B Degroote RL Witte T Deeg CA Aberrant Migratory Behavior of Immune Cells in Recurrent Autoimmune Uveitis in Horses. Front Cell Dev Biol (2020) 8:101. 10.3389/fcell.2020.00101 32211402 PMC7076317

[37] Tooley AJ Gilden J Jacobelli J Beemiller P Trimble WS Kinoshita M Amoeboid T lymphocytes require the septin cytoskeleton for cortical integrity and persistent motility. Nat Cell Biol (2009) 11(1):17–26. 10.1038/ncb1808 ncb1808 [pii]. 19043408 PMC3777658

[38] Aydin SE Kilic SS Aytekin C Kumar A Porras O Kainulainen L DOCK8 deficiency: clinical and immunological phenotype and treatment options - a review of 136 patients. J Clin Immunol (2015) 35(2):189–98. 10.1007/s10875-014-0126-0 25627830

[39] Saldinger LK Nelson SG Bellone RR Lassaline M Mack M Walker NJ Horses with equine recurrent uveitis have an activated CD4+ T-cell phenotype that can be modulated by mesenchymal stem cells in vitro. Vet Ophthalmol (2020) 23(1):160–70. 10.1111/vop.12704 PMC6980227 31441218

[40] Curto E Messenger KM Salmon JH Gilger BC Cytokine and chemokine profiles of aqueous humor and serum in horses with uveitis measured using multiplex bead immunoassay analysis. Vet Immunol Immunopathol (2016) 182:43–51. 10.1016/j.vetimm.2016.09.008 27863549

[41] Luger D Silver PB Tang J Cua D Chen Z Iwakura Y Either a Th17 or a Th1 effector response can drive autoimmunity: conditions of disease induction affect dominant effector category. J Exp Med (2008) 205(4):799–810. 10.1084/jem.20071258 jem.20071258 [pii]. 18391061 PMC2292220

[42] Regan DP Aarnio MC Davis WS Carmichael KP Vandenplas ML Lauderdale JD Characterization of cytokines associated with Th17 cells in the eyes of horses with recurrent uveitis. Vet Ophthalmol (2012) 15(3):145–52. 10.1111/j.1463-5224.2011.00951.x 22051225

[43] Simeonova GP Krastev SZ Simeonov RS Immunological and pathological investigations in equine experimental uveitis. Vet Res Commun (2016) 40(3-4):107–15. 10.1007/s11259-016-9659-4 27344152

[44] Sharma A Rudra D Emerging Functions of Regulatory T Cells in Tissue Homeostasis. Front Immunol (2018) 9:883. 10.3389/fimmu.2018.00883 29887862 PMC5989423

[45] Silver PB Horai R Chen J Jittayasothorn Y Chan CC Villasmil R Retina-specific T regulatory cells bring about resolution and maintain remission of autoimmune uveitis. J Immunol (2015) 194(7):3011–9. 10.4049/jimmunol.1402650 PMC4459505 25716996

[46] Gilbert RM Zhang X Sampson RD Ehrenstein MR Nguyen DX Chaudhry M Clinical Remission of Sight-Threatening Non-Infectious Uveitis Is Characterized by an Upregulation of Peripheral T-Regulatory Cell Polarized Towards T-bet and TIGIT. Front Immunol (2018) 9:907. 10.3389/fimmu.2018.00907 29774027 PMC5943505

[47] Ruggieri S Frassanito MA Dammacco R Guerriero S Treg lymphocytes in autoimmune uveitis. Ocul Immunol Inflammation (2012) 20(4):255–61. 10.3109/09273948.2012.681830 22564107

[48] Yeh S Li Z Forooghian F Hwang FS Cunningham MA Pantanelli S CD4+Foxp3+ T-regulatory cells in noninfectious uveitis. Arch Ophthalmol (2009) 127(4):407–13. 10.1001/archophthalmol.2009.32 PMC2928652 19365016

[49] Horai R Zarate-Blades CR Dillenburg-Pilla P Chen J Kielczewski JL Silver PB Microbiota-Dependent Activation of an Autoreactive T Cell Receptor Provokes Autoimmunity in an Immunologically Privileged Site. Immunity (2015) 43(2):343–53. 10.1016/j.immuni.2015.07.014 PMC4544742 26287682

[50] Horai R Caspi RR Microbiome and Autoimmune Uveitis. Front Immunol (2019) 10:232. 10.3389/fimmu.2019.00232 30837991 PMC6389708

[51] Ye Z Wu C Zhang N Du L Cao Q Huang X Altered gut microbiome composition in patients with Vogt-Koyanagi-Harada disease. Gut Microbes (2020) 11(3):539–55. 10.1080/19490976.2019.1700754 PMC7524263 31928124

[52] Shimizu J Kubota T Takada E Takai K Fujiwara N Arimitsu N Bifidobacteria Abundance-Featured Gut Microbiota Compositional Change in Patients with Behcet’s Disease. PloS One (2016) 11(4):e0153746. 10.1371/journal.pone.0153746 27105322 PMC4841557

[53] MacDonald ES Barrett JG The Potential of Mesenchymal Stem Cells to Treat Systemic Inflammation in Horses. Front Vet Sci (2019) 6:507. 10.3389/fvets.2019.00507 32039250 PMC6985200

[54] Davis AB Schnabel LV Gilger BC Subconjunctival bone marrow-derived mesenchymal stem cell therapy as a novel treatment alternative for equine immune-mediated keratitis: A case series. Vet Ophthalmol (2019) 22(5):674–82. 10.1111/vop.12641 30715781

[55] Werry H Gerhards H [The surgical therapy of equine recurrent uveitis]. Tierarztl Prax (1992) 20(2):178–86. 1609401

[56] Gilger BC Salmon JH Wilkie DA Cruysberg LP Kim J Hayat M A novel bioerodible deep scleral lamellar cyclosporine implant for uveitis. Invest Ophthalmol Vis Sci (2006) 47(6):2596–605. 10.1167/iovs.05-1540 47/6/2596 [pii]. 16723476

[57] Pepple KL Wilson L Van Gelder RN Comparison of Aqueous and Vitreous Lymphocyte Populations From Two Rat Models of Experimental Uveitis. Invest Ophthalmol Vis Sci (2018) 59(6):2504–11. 10.1167/iovs.18-24192 PMC5963002 29847657

[58] Kerr EC Raveney BJ Copland DA Dick AD Nicholson LB Analysis of retinal cellular infiltrate in experimental autoimmune uveoretinitis reveals multiple regulatory cell populations. J Autoimmun (2008) 31(4):354–61. 10.1016/j.jaut.2008.08.006 18838247

[59] Jones LS Rizzo LV Agarwal RK Tarrant TK Chan CC Wiggert B IFN-gamma-deficient mice develop experimental autoimmune uveitis in the context of a deviant effector response. J Immunol (1997) 158(12):5997–6005. 9190954

[60] Kim SJ Zhang M Vistica BP Chan CC Shen DF Wawrousek EF Induction of ocular inflammation by T-helper lymphocytes type 2. Invest Ophthalmol Vis Sci (2002) 43(3):758–65. 11867595

[61] Zipplies JK Kirschfink M Amann B Hauck SM Stangassinger M Deeg CA Complement factor B expression profile in a spontaneous uveitis model. Immunobiology (2010) 215(12):949–55. 10.1016/j.imbio.2010.02.003 20334949

[62] Robertson MJ Erwig LP Liversidge J Forrester JV Rees AJ Dick AD Retinal microenvironment controls resident and infiltrating macrophage function during uveoretinitis. Invest Ophthalmol Vis Sci (2002) 43(7):2250–7. 12091424

[63] Khera TK Copland DA Boldison J Lait PJ Szymkowski DE Dick AD Tumour necrosis factor-mediated macrophage activation in the target organ is critical for clinical manifestation of uveitis. Clin Exp Immunol (2012) 168(2):165–77. 10.1111/j.1365-2249.2012.04567.x PMC3390517 22471277

[64] Chinnery HR McMenamin PG Dando SJ Macrophage physiology in the eye. Pflugers Arch (2017) 469(3-4):501–15. 10.1007/s00424-017-1947-5 28233124

[65] Okunuki Y Mukai R Nakao T Tabor SJ Butovsky O Dana R Retinal microglia initiate neuroinflammation in ocular autoimmunity. Proc Natl Acad Sci U.S.A. (2019) 116(20):9989–98. 10.1073/pnas.1820387116 PMC6525481 31023885

[66] Degroote RL Hauck SM Kremmer E Amann B Ueffing M Deeg CA Altered expression of talin 1 in peripheral immune cells points to a significant role of the innate immune system in spontaneous autoimmune uveitis. J Proteomics (2012) 75(14):4536–44. 10.1016/j.jprot.2012.01.023 22306886

[67] Weigand M Hauck SM Deeg CA Degroote RL Deviant proteome profile of equine granulocytes associates to latent activation status in organ specific autoimmune disease. J Proteomics (2020) 230:1–9. 10.1016/j.jprot.2020.103989 32977044

[68] Naegele M Tillack K Reinhardt S Schippling S Martin R Sospedra M Neutrophils in multiple sclerosis are characterized by a primed phenotype. J Neuroimmunol (2012) 242(1-2):60–71. 10.1016/j.jneuroim.2011.11.009 22169406

[69] Degroote RL Weigand M Hauck SM Deeg CA IL8 and PMA Trigger the Regulation of Different Biological Processes in Granulocyte Activation. Front Immunol (2019) 10:3064. 10.3389/fimmu.2019.03064 32010136 PMC6973177

[70] Fingerhut L Ohnesorge B von Borstel M Schumski A Strutzberg-Minder K Morgelin M Neutrophil Extracellular Traps in the Pathogenesis of Equine Recurrent Uveitis (ERU). Cells (2019) 8(12):1–22. 10.3390/cells8121528 PMC6953072 31783639

[71] Zipplies JK Hauck SM Schoeffmann S Amann B van der Meijden CH Stangassinger M Kininogen in autoimmune uveitis: decrease in peripheral blood stream versus increase in target tissue. Invest Ophthalmol Vis Sci (2010) 51(1):375–82. 10.1167/iovs.09-4094 19696180

[72] Natoli R Fernando N Jiao H Racic T Madigan M Barnett NL Retinal Macrophages Synthesize C3 and Activate Complement in AMD and in Models of Focal Retinal Degeneration. Invest Ophthalmol Vis Sci (2017) 58(7):2977–90. 10.1167/iovs.17-21672 28605809

[73] Chen M Muckersie E Robertson M Forrester JV Xu H Up-regulation of complement factor B in retinal pigment epithelial cells is accompanied by complement activation in the aged retina. Exp Eye Res (2008) 87(6):543–50. 10.1016/j.exer.2008.09.005 18926817

[74] Mohlin C Sandholm K Ekdahl KN Nilsson B The link between morphology and complement in ocular disease. Mol Immunol (2017) 89:84–99. 10.1016/j.molimm.2017.05.028 28622910

[75] Bora NS Jha P Bora PS The role of complement in ocular pathology. Semin Immunopathol (2008) 30(2):85–95. 10.1007/s00281-008-0110-y 18299835 PMC2315691

[76] Zhang L Bell BA Yu M Chan CC Peachey NS Fung J Complement anaphylatoxin receptors C3aR and C5aR are required in the pathogenesis of experimental autoimmune uveitis. J Leukoc Biol (2016) 99(3):447–54. 10.1189/jlb.3A0415-157R PMC6608042 26394814

[77] Jha P Sohn JH Xu Q Wang Y Kaplan HJ Bora PS Suppression of complement regulatory proteins (CRPs) exacerbates experimental autoimmune anterior uveitis (EAAU). J Immunol (2006) 176(12):7221–31. 10.4049/jimmunol.176.12.7221 16751365

[78] Jha P Sohn JH Xu Q Nishihori H Wang Y Nishihori S The complement system plays a critical role in the development of experimental autoimmune anterior uveitis. Invest Ophthalmol Vis Sci (2006) 47(3):1030–8. 10.1167/iovs.05-1062 PMC1975680 16505038

[79] Chen M Muckersie E Luo C Forrester JV Xu H Inhibition of the alternative pathway of complement activation reduces inflammation in experimental autoimmune uveoretinitis. Eur J Immunol (2010) 40(10):2870–81. 10.1002/eji.201040323 20806290

[80] Chiaradia E Miller I In slow pace towards the proteome of equine body fluids. J Proteomics (2020) 225:103880. 10.1016/j.jprot.2020.103880 32569818

[81] Zipplies JK Hauck SM Eberhardt C Hirmer S Amann B Stangassinger M Miscellaneous vitreous-derived IgM antibodies target numerous retinal proteins in equine recurrent uveitis. Vet Ophthalmol (2012) 15 Suppl;2:57–64. 10.1111/j.1463-5224.2012.01010.x 22432720

[82] Zipplies JK Hauck SM Schoeffmann S Amann B Stangassinger M Ueffing M Serum PEDF levels are decreased in a spontaneous animal model for human autoimmune uveitis. J Proteome Res (2009) 8(2):992–8. 10.1021/pr800694y 10.1021/pr800694y [pii]. 19113885

[83] Guo YL Colman RW Two faces of high-molecular-weight kininogen (HK) in angiogenesis: bradykinin turns it on and cleaved HK (HKa) turns it off. J Thromb Haemost (2005) 3(4):670–6. 10.1111/j.1538-7836.2005.01218.x 15733059

[84] Gobel K Asaridou CM Merker M Eichler S Herrmann AM Geuss E Plasma kallikrein modulates immune cell trafficking during neuroinflammation via PAR2 and bradykinin release. Proc Natl Acad Sci USA (2019) 116(1):271–6. 10.1073/pnas.1810020116 PMC6320546 30559188

[85] Zhang SX Wang JJ Gao G Shao C Mott R Ma JX Pigment epithelium-derived factor (PEDF) is an endogenous antiinflammatory factor. FASEB J (2006) 20(2):323–5. 10.1096/fj.05-4313fje 05-4313fje [pii]. 16368716

[86] Comitato A Subramanian P Turchiano G Montanari M Becerra SP Marigo V Pigment epithelium-derived factor hinders photoreceptor cell death by reducing intracellular calcium in the degenerating retina. Cell Death Dis (2018) 9(5):560. 10.1038/s41419-018-0613-y 29752430 PMC5948223

[87] Eichler W Savkovic-Cvijic H Burger S Beck M Schmidt M Wiedemann P Muller Cell-Derived PEDF Mediates Neuroprotection via STAT3 Activation. Cell Physiol Biochem (2017) 44(4):1411–24. 10.1159/000485537 29186716

[88] Wang X Liu X Ren Y Liu Y Han S Zhao J PEDF protects human retinal pigment epithelial cells against oxidative stress via upregulation of UCP2 expression. Mol Med Rep (2019) 19(1):59–74. 10.3892/mmr.2018.9645 30431098 PMC6297793

[89] Strauss O The retinal pigment epithelium in visual function. Physiol Rev (2005) 85(3):845–81. 10.1152/physrev.00021.2004 15987797

[90] Szober CM Hauck SM Euler KN Frohlich KJ Alge-Priglinger C Ueffing M Profound re-organization of cell surface proteome in equine retinal pigment epithelial cells in response to in vitro culturing. Int J Mol Sci (2012) 13(11):14053–72. 10.3390/ijms131114053 PMC3509565 23203049

[91] Uhl PB Szober CM Amann B Alge-Priglinger C Ueffing M Hauck SM In situ cell surface proteomics reveals differentially expressed membrane proteins in retinal pigment epithelial cells during autoimmune uveitis. J Proteomics (2014) 109:50–62. 10.1016/j.jprot.2014.06.020 24998980

[92] Uhl PB Amann B Hauck SM Deeg CA Novel localization of peripherin 2, the photoreceptor-specific retinal degeneration slow protein, in retinal pigment epithelium. Int J Mol Sci (2015) 16(2):2678–92. 10.3390/ijms16022678 PMC4346858 25629227

[93] Xu H Forrester JV Liversidge J Crane IJ Leukocyte trafficking in experimental autoimmune uveitis: breakdown of blood-retinal barrier and upregulation of cellular adhesion molecules. Invest Ophthalmol Vis Sci (2003) 44(1):226–34. 10.1167/iovs.01-1202 12506079

[94] Xu H Manivannan A Liversidge J Sharp PF Forrester JV Crane IJ Involvement of CD44 in leukocyte trafficking at the blood-retinal barrier. J Leukoc Biol (2002) 72:1133–41, 72(6). 12488494

[95] Xu H Manivannan A Goatman KA Jiang HR Liversidge J Sharp PF Reduction in shear stress, activation of the endothelium, and leukocyte priming are all required for leukocyte passage across the blood–retina barrier. J Leukoc Biol (2004) 75(2):224–32. 10.1189/jlb.1002479 14634055

[96] Xu H Manivannan A Jiang HR Liversidge J Sharp PF Forrester JV Recruitment of IFN-gamma-producing (Th1-like) cells into the inflamed retina in vivo is preferentially regulated by P-selectin glycoprotein ligand 1:P/E-selectin interactions. J Immunol (2004) 172(5):3215–24. 10.4049/jimmunol.172.5.3215 14978129

[97] Crane IJ Xu H Wallace C Manivannan A Mack M Liversidge J Involvement of CCR5 in the passage of Th1-type cells across the blood-retina barrier in experimental autoimmune uveitis. J Leukoc Biol (2006) 79(3):435–43. 10.1189/jlb.0305130 16365158

[98] Thurau SR Mempel TR Flugel A Diedrichs-Mohring M Krombach F Kawakami N The fate of autoreactive, GFP+ T cells in rat models of uveitis analyzed by intravital fluorescence microscopy and FACS. Int Immunol (2004) 16(11):1573–82. 10.1093/intimm/dxh158 15351788

[99] Zhao J Chen M Xu H Experimental autoimmune uveoretinitis (EAU)-related tissue damage and angiogenesis is reduced in CCL2(-)/(-)CX(3)CR1gfp/gfp mice. Invest Ophthalmol Vis Sci (2014) 55(11):7572–82. 10.1167/iovs.14-15495 25342612

[100] Chen M Zhao J Ali IHA Marry S Augustine J Bhuckory M Cytokine Signaling Protein 3 Deficiency in Myeloid Cells Promotes Retinal Degeneration and Angiogenesis through Arginase-1 Up-Regulation in Experimental Autoimmune Uveoretinitis. Am J Pathol (2018) 188(4):1007–20. 10.1016/j.ajpath.2017.12.021 29452101

[101] Amadi-Obi A Yu CR Liu X Mahdi RM Clarke GL Nussenblatt RB TH17 cells contribute to uveitis and scleritis and are expanded by IL-2 and inhibited by IL-27/STAT1. Nat Med (2007) 13(6):711–8. 10.1038/nm1585 nm1585 [pii]. 17496900

[102] Bing SJ Shemesh I Chong WP Horai R Jittayasothorn Y Silver PB AS101 ameliorates experimental autoimmune uveitis by regulating Th1 and Th17 responses and inducing Treg cells. J Autoimmun (2019) 100:52–61. 10.1016/j.jaut.2019.02.006 30853312 PMC6513711

[103] Horai R Caspi RR Cytokines in autoimmune uveitis. J Interferon Cytokine Res (2011) 31(10):733–44. 10.1089/jir.2011.0042 PMC3189550 21787221

[104] Weinstein JE Pepple KL Cytokines in uveitis. Curr Opin Ophthalmol (2018) 29(3):267–74. 10.1097/ICU.0000000000000466 PMC7199509 29521875

[105] Hauck SM Dietter J Kramer RL Hofmaier F Zipplies JK Amann B Deciphering membrane-associated molecular processes in target tissue of autoimmune uveitis by label-free quantitative mass spectrometry. Mol Cell Proteomics (2010) 9(10):2292–305. 10.1074/mcp.M110.001073 PMC2953921 20601722

[106] Newman E Reichenbach A The Muller cell: a functional element of the retina. Trends Neurosci (1996) 19(8):307–12. 10.1016/0166-2236(96)10040-0 8843598

[107] Eberhardt C Amann B Feuchtinger A Hauck SM Deeg CA Differential expression of inwardly rectifying K+ channels and aquaporins 4 and 5 in autoimmune uveitis indicates misbalance in Muller glial cell-dependent ion and water homeostasis. Glia (2011) 59(5):697–707. 10.1002/glia.21139 21305615

[108] Bringmann A Pannicke T Grosche J Francke M Wiedemann P Skatchkov SN Muller cells in the healthy and diseased retina. Prog Retin Eye Res (2006) 25(4):397–424. 10.1016/j.preteyeres.2006.05.003 16839797

[109] Bringmann A Iandiev I Pannicke T Wurm A Hollborn M Wiedemann P Cellular signaling and factors involved in Muller cell gliosis: neuroprotective and detrimental effects. Prog Retin Eye Res (2009) 28(6):423–51. 10.1016/j.preteyeres.2009.07.001 19660572

[110] Deeg CA Amann B Lutz K Hirmer S Lutterberg K Kremmer E Aquaporin 11, a regulator of water efflux at retinal Muller glial cell surface decreases concomitant with immune-mediated gliosis. J Neuroinflammation (2016) 13(1):89. 10.1186/s12974-016-0554-2 27107718 PMC4842293

[111] Hauck SM Hofmaier F Dietter J Swadzba ME Blindert M Amann B Label-free LC-MSMS analysis of vitreous from autoimmune uveitis reveals a significant decrease in secreted Wnt signalling inhibitors DKK3 and SFRP2. J Proteomics (2012) 75(14):4545–54. 10.1016/j.jprot.2012.04.052 22634081

[112] Shi J Chi S Xue J Yang J Li F Liu X Emerging Role and Therapeutic Implication of Wnt Signaling Pathways in Autoimmune Diseases. J Immunol Res (2016) 2016:9392132. 10.1155/2016/9392132 27110577 PMC4826689

[113] Cici D Corrado A Rotondo C Cantatore FP Wnt Signaling and Biological Therapy in Rheumatoid Arthritis and Spondyloarthritis. Int J Mol Sci (2019) 20(22):1–15. 10.3390/ijms20225552 PMC6888549 31703281

[114] Deeg CA Eberhardt C Hofmaier F Amann B Hauck SM Osteopontin and fibronectin levels are decreased in vitreous of autoimmune uveitis and retinal expression of both proteins indicates ECM re-modeling. PloS One (2011) 6(12):e27674. 10.1371/journal.pone.0027674 22194789 PMC3237414

[115] Pankov R Yamada KM Fibronectin at a glance. J Cell Sci (2002) 115(Pt 20):3861–3. 10.1242/jcs.00059 12244123

[116] Wang XL Yu T Zhang JS Yan QC Luo YH Fibronectin and focal adhesion kinase small interfering RNA modulate rat retinal Muller cells adhesion and migration. Cell Mol Neurobiol (2009) 29(4):549–56. 10.1007/s10571-009-9346-x PMC11505797 19172391

[117] Braitch M Constantinescu CS The role of osteopontin in experimental autoimmune encephalomyelitis (EAE) and multiple sclerosis (MS). Inflammation Allergy Drug Targets (2010) 9(4):249–56. 10.2174/187152810793358778 20887272

[118] Hikita ST Vistica BP Jones HR Keswani JR Watson MM Ericson VR Osteopontin is proinflammatory in experimental autoimmune uveitis. Invest Ophthalmol Vis Sci (2006) 47(10):4435–43. 10.1167/iovs.06-0064 17003437

[119] Kitamura M Iwabuchi K Kitaichi N Kon S Kitamei H Namba K Osteopontin aggravates experimental autoimmune uveoretinitis in mice. J Immunol (2007) 178(10):6567–72. 10.4049/jimmunol.178.10.6567 17475887

[120] Del Rio P Irmler M Arango-Gonzalez B Favor J Bobe C Bartsch U GDNF-induced osteopontin from Muller glial cells promotes photoreceptor survival in the Pde6brd1 mouse model of retinal degeneration. Glia (2011) 59(5):821–32. 10.1002/glia.21155 21360756

[121] Ruzafa N Pereiro X Lepper MF Hauck SM Vecino E A Proteomics Approach to Identify Candidate Proteins Secreted by Muller Glia that Protect Ganglion Cells in the Retina. Proteomics (2018) 18(11):e1700321. 10.1002/pmic.201700321 29645351

[122] Hofmaier F Hauck SM Amann B Degroote RL Deeg CA Changes in matrix metalloproteinase network in a spontaneous autoimmune uveitis model. Invest Ophthalmol Vis Sci (2011) 52(5):2314–20. 10.1167/iovs.10-6475 21228380

[123] Brew K Dinakarpandian D Nagase H Tissue inhibitors of metalloproteinases: evolution, structure and function. Biochim Biophys Acta (2000) 1477(1-2):267–83. 10.1016/s0167-4838(99)00279-4 10708863

[124] Murphy G Nagase H Progress in matrix metalloproteinase research. Mol Aspects Med (2008) 29(5):290–308. 10.1016/j.mam.2008.05.002 18619669 PMC2810947

[125] Magnoni S Baker A Thomson S Jordan G George SJ McColl BW Neuroprotective effect of adenoviral-mediated gene transfer of TIMP-1 and -2 in ischemic brain injury. Gene Ther (2007) 14(7):621–5. 10.1038/sj.gt.3302894 17235293

[126] Woods CC Sundar K Tessler C Lebsack TW Grainger L Nielsen A Tissue inhibitor of metalloproteinase-2 inhibits T-cell infiltration and preserves pancreatic beta-cell function in an in vitro type 1 diabetes mellitus model. J Autoimmun (2006) 27(1):28–37. 10.1016/j.jaut.2006.04.004 16765565

[127] Abraham M Shapiro S Karni A Weiner HL Miller A Gelatinases (MMP-2 and MMP-9) are preferentially expressed by Th1 vs. Th2 cells. Cells J Neuroimmunol (2005) 163(1-2):157–64. 10.1016/j.jneuroim.2005.02.001 15885317

[128] Wu B Crampton SP Hughes CC Wnt signaling induces matrix metalloproteinase expression and regulates T cell transmigration. Immunity (2007) 26(2):227–39. 10.1016/j.immuni.2006.12.007 PMC1855210 17306568

[129] El-Shabrawi Y Walch A Hermann J Egger G Foster CS Inhibition of MMP-dependent chemotaxis and amelioration of experimental autoimmune uveitis with a selective metalloproteinase-2 and -9 inhibitor. J Neuroimmunol (2004) 155(1-2):13–20. 10.1016/j.jneuroim.2004.05.010 15342192

[130] Swadzba ME Hirmer S Amann B Hauck SM Deeg CA Vitreal IgM autoantibodies target neurofilament medium in a spontaneous model of autoimmune uveitis. Invest Ophthalmol Vis Sci (2012) 53(1):294–300. 10.1167/iovs.11-8734 22199250

[131] Swadzba ME Hauck SM Naim HY Amann B Deeg CA Retinal glycoprotein enrichment by concanavalin a enabled identification of novel membrane autoantigen synaptotagmin-1 in equine recurrent uveitis. PloS One (2012) 7(12):e50929. 10.1371/journal.pone.0050929 23236410 PMC3517615

[132] Kalsow CM Dubielzig RR Dwyer AE Immunopathology of pineal glands from horses with uveitis. Invest Ophthalmol Vis Sci (1999) 40(7):1611–5. 10359346

[133] Eberhardt C Amann B Stangassinger M Hauck SM Deeg CA Isolation, characterization and establishment of an equine retinal glial cell line: a prerequisite to investigate the physiological function of Muller cells in the retina. J Anim Physiol Anim Nutr (Berl) (2012) 96(2):260–9. 10.1111/j.1439-0396.2011.01147.x 21535230

